# Contrast-Enhanced Ultrasound Combined with the NLR–PMR Phenotype for Prostate Cancer Prediction and Risk Stratification in Patients with PSA 4–20 ng/mL and PI-RADS ≤ 3

**DOI:** 10.3390/diagnostics16213680

**Published:** 2026-11-07

**Authors:** Wanlin Zhou, Zhenfeng Ding, Yurou Guo, Juanyi Han, Huijia Zhao, Xin Bai, Wen Cheng, Bolin Wu

**Affiliations:** 1 Department of Ultrasound, Harbin Medical University Cancer Hospital, Harbin 150006, China; zhouwanlin@hrbmu.edu.cn (W.Z.); 13544042167@163.com (Z.D.); hmuhjy9387@163.com (J.H.); zhaohuijia0122@163.com (H.Z.); baixin@hrbmu.edu.cn (X.B.); 2 College of Bioinformatics Science and Technology, Harbin Medical University, Harbin 150081, China; gm20190130@163.com

**Keywords:** prostate cancer, clinically significant prostate cancer, contrast-enhanced ultrasound, inflammatory phenotype, PI-RADS, risk stratification

## Abstract

**Background/Objectives**: To assess contrast-enhanced ultrasound (CEUS) combined with the neutrophil-to-lymphocyte ratio–platelet-to-monocyte ratio (NLR–PMR) phenotype for predicting prostate cancer (PCa) and clinically significant PCa (csPCa) in patients with PSA 4–20 ng/mL and PI-RADS ≤ 3. **Methods**: This single-center retrospective study included 418 biopsy patients. Outcomes were PCa and csPCa (ISUP Grade Group ≥ 2). A continuous NLR–PMR score and CEUS were added to an age–PSA density baseline model. Discrimination was assessed using the area under the receiver operating characteristic curve (AUC). Three risk strata were defined using CEUS and NLR–PMR status. **Results**: PCa and csPCa were detected in 60 (14.4%) and 42 (10.0%) patients. Adding NLR–PMR improved AUCs beyond age and PSA density for PCa (0.798 vs. 0.747; *p* = 0.025) and csPCa (0.805 vs. 0.752; *p* = 0.026). Combined-model AUCs were 0.798 for PCa and 0.812 for csPCa. CEUS positivity remained associated with csPCa after adjustment (OR = 2.26, 95% CI: 1.08–4.91; *p* = 0.033). Across low-, intermediate-, and high-risk strata, detection rates were 3.9%, 15.2%, and 34.3% for PCa and 2.0%, 8.0%, and 28.4% for csPCa. The low-risk stratum comprised 48.8% of patients, with a csPCa negative predictive value of 98.0% and no ISUP Grade Group 4–5 cancers. Dual CEUS/NLR–PMR positivity increased with pathological grade (*p* < 0.001). **Conclusions**: In this exploratory cohort, NLR–PMR improved discrimination for PCa and csPCa, and CEUS further differentiated csPCa risk among NLR–PMR-positive patients. The combined assessment identified strata with progressively increasing cancer detection rates, providing preliminary evidence for individualized pre-biopsy risk assessment. Prospective multicenter external validation is required before clinical implementation.

## 1. Introduction

Serum prostate-specific antigen (PSA) is widely used for the early detection of prostate cancer (PCa), but its limited tumor specificity necessitates integration with other risk information [1]. Multiparametric magnetic resonance imaging (mpMRI) has improved pre-biopsy risk assessment and lesion targeting [2]. Nevertheless, clinically significant prostate cancer (csPCa) cannot be completely excluded in patients with Prostate Imaging Reporting and Data System (PI-RADS) scores ≤ 3 [3]. A large multi-institutional cohort reported a csPCa detection rate of approximately 20% among patients with PI-RADS 3 lesions [4]. Although PSA density (PSAD) can further refine risk assessment in these patients, reliance on a single threshold may reduce biopsies at the cost of missing some csPCa cases [5]. Moreover, the predictive performance and optimal threshold of PSAD vary according to prostate volume and PI-RADS category [6]. Additional information is therefore needed to improve biopsy decision-making in patients with elevated PSA levels but no highly suspicious lesions on MRI. The EAU guideline update recommends integrating MRI findings with PSAD and other clinical risk factors when considering biopsy, including in patients with negative or equivocal MRI [7]. Comparative evidence from Ahdoot et al. also illustrates how systematic and MRI-targeted sampling provide complementary diagnostic information and influence the reference standard used to assess risk models [8].

Contrast-enhanced ultrasound (CEUS) enables real-time visualization of prostatic microvascular perfusion and provides qualitative and quantitative information for lesion assessment [9]. The EFSUMB recommendations provide a technical framework for non-hepatic CEUS, including contrast administration, low-mechanical-index imaging, and acquisition of enhancement information; consistent acquisition and interpretation are important for reproducibility [10]. Previous research has suggested that adding CEUS to PI-RADS may improve pre-biopsy risk assessment in patients with PSA levels of 4–10 ng/mL [11]. However, benign prostatic hyperplasia and chronic inflammation may also exhibit abnormal enhancement patterns, limiting the diagnostic specificity of CEUS [12]. Multiparametric ultrasound approaches may improve lesion characterization by integrating complementary imaging features [13]. More recently, the combination of hemodynamic and tissue-stiffness features has shown improved performance in predicting csPCa [14]. These findings suggest that combining CEUS-derived perfusion information with other risk factors may enhance pre-biopsy assessment.

Blood-based inflammatory markers derived from routine complete blood counts are readily available, inexpensive, and reproducible. A systematic review and meta-analysis by Salciccia et al. identified heterogeneous prognostic findings for NLR and platelet-to-lymphocyte ratio across PCa settings [15]. This evidence supports studying inflammatory markers while highlighting that prognostic associations require separate evaluation for pre-biopsy diagnosis. Various hematological inflammatory markers have been evaluated in relation to prostate biopsy outcomes, but their independent predictive ability and incremental value have been inconsistent [16]. The neutrophil-to-lymphocyte ratio (NLR) has previously been combined with PI-RADS in pre-biopsy prediction models for csPCa [17]. In patients with PSA levels of 4–20 ng/mL, composite indices such as the pan-immune-inflammation value have also shown discriminatory value for PCa [18]. Associations between monocyte-based ratios and biopsy-detected csPCa have also been reported [19]. By incorporating different circulating blood-cell components, NLR and the platelet-to-monocyte ratio (PMR) may provide complementary inflammation-related information. Combining these hematological features with local perfusion information from CEUS may enable a more comprehensive risk assessment in patients with elevated PSA levels and low-suspicion MRI findings; however, evidence supporting this approach remains limited.

Accordingly, this study focused on patients with PSA levels of 4–20 ng/mL and PI-RADS scores ≤ 3, in whom uncertainty about cancer risk may persist after PSA testing and MRI. We investigated the associations of qualitative CEUS findings and the NLR–PMR phenotype with biopsy-detected PCa and csPCa, and evaluated their combined performance in prediction and risk stratification. The aim was to explore whether these complementary sources of information could help refine pre-biopsy risk assessment and inform individualized biopsy decisions.

## 2. Materials and Methods

### 2.1. Study Population

**Figure 1:**
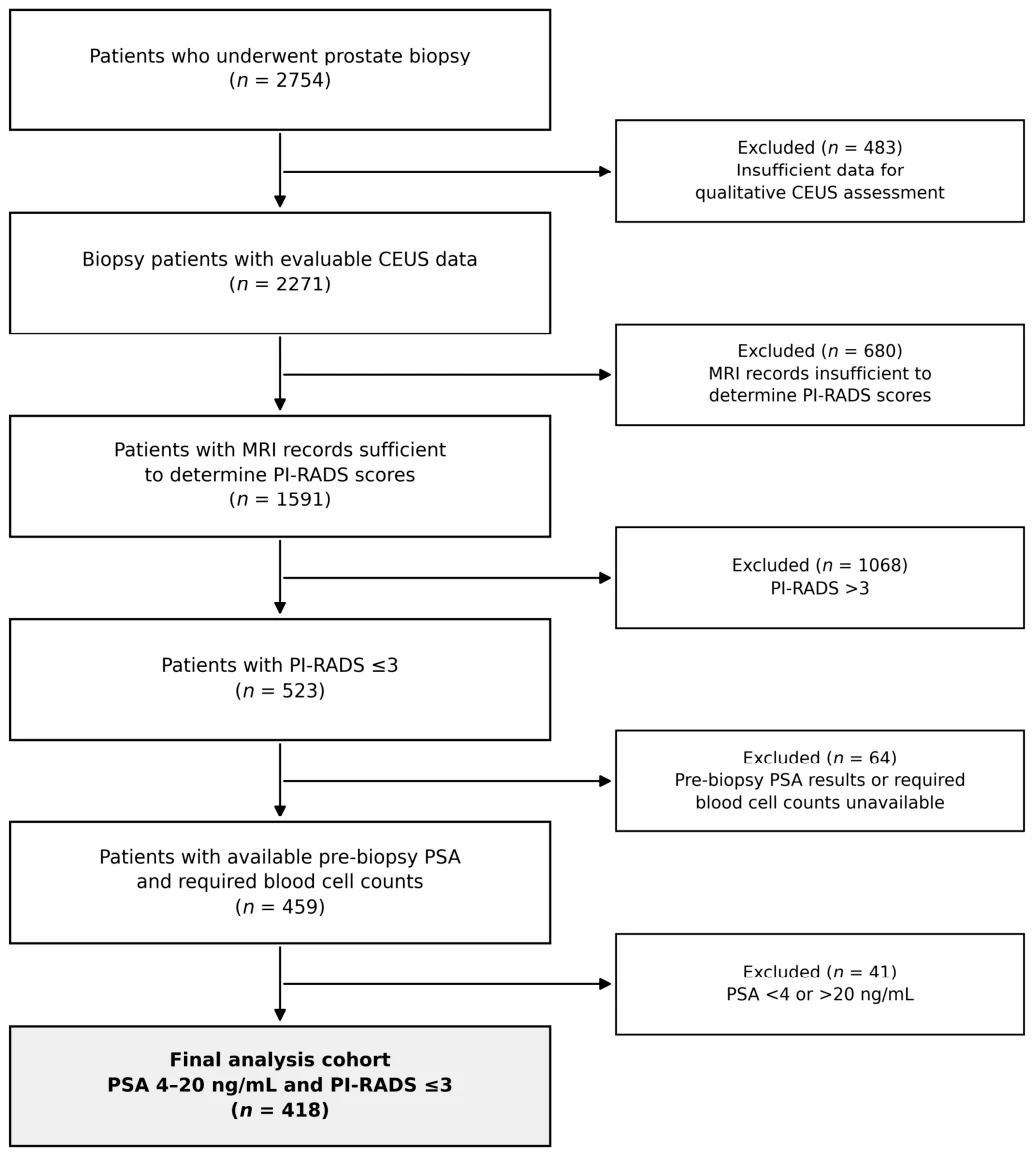
Patient screening and analysis workflow. During the study period, 2754 patients underwent prostate biopsy. Sequential screening according to CEUS evaluability, MRI availability and PI-RADS score, availability of PSA and required blood cell counts, and PSA range yielded 418 patients for analysis. Abbreviations: CEUS, contrast-enhanced ultrasound; PI-RADS, Prostate Imaging Reporting and Data System; PSA, prostate-specific antigen.

This single-center retrospective study reviewed 2754 patients who underwent prostate biopsy at Harbin Medical University Cancer Hospital between May 2020 and June 2026. Eligible patients had pre-biopsy PSA levels of 4–20 ng/mL, PI-RADS v2.1 scores ≤ 3, evaluable CEUS data, definitive pathology from a 12-core biopsy, and sufficient clinical and laboratory data. Patients with previous PCa or related treatment were excluded. The final cohort included 418 patients; the sequential exclusion reasons and counts are shown in Figure 1. Biopsy decisions were made by the treating clinician after considering additional clinical indications; biopsy was performed selectively among patients meeting the PSA and MRI criteria.

This study was approved by the Ethics Committee of Harbin Medical University Cancer Hospital.

### 2.2. MRI Assessment

All patients underwent 3.0-T prostate mpMRI before biopsy using either a MAGNETOM Skyra scanner (Siemens Healthineers, Erlangen, Germany) or a SIGNA Architect scanner (GE Healthcare, Waukesha, WI, USA). The routine imaging protocol included T2-weighted imaging (T2WI), diffusion-weighted imaging (DWI), and dynamic contrast-enhanced MRI (DCE-MRI). Two radiologists with at least 5 years of experience in prostate MRI independently assigned PI-RADS scores according to PI-RADS version 2.1 while blinded to the patients’ clinical information and final biopsy results [20]. Any disagreement was reviewed by a third senior radiologist and resolved by consensus.

### 2.3. CEUS Examination and Interpretation

All patients underwent conventional transrectal ultrasound and CEUS before biopsy. Examinations were performed using either a MyLabTwice ultrasound system with a TRT-33 transrectal probe operating at 3.0–13.0 MHz (Esaote, Genoa, Italy) or a Resona R9 Exp system with an ELC13-4U transrectal probe operating at 4.0–13.0 MHz (Mindray, Shenzhen, China). In low-mechanical-index mode (MI < 0.1), a 2.4mL bolus of sulfur hexafluoride microbubble contrast agent (SonoVue; Bracco, Milan, Italy) was administered through an antecubital vein, followed by a rapid 5mL saline flush. Prostatic perfusion was continuously observed for 120 s after injection, and dynamic video clips were stored.

**Figure 2:**
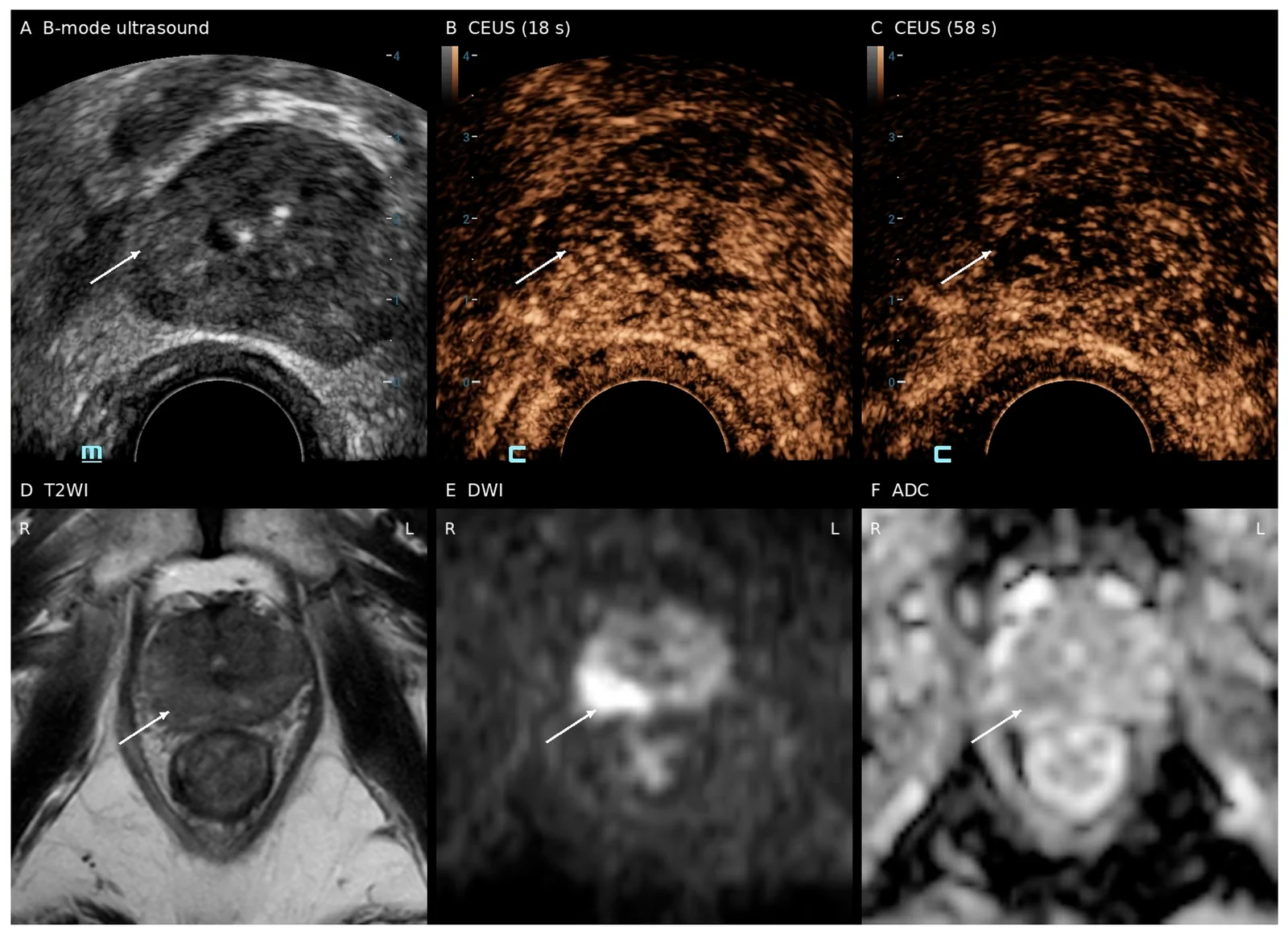
Ultrasound and MRI findings in a representative patient with a PI-RADS 3 lesion and biopsy-confirmed clinically significant prostate cancer. (**A**) Transrectal B-mode ultrasound shows the right peripheral-zone lesion region. (**B**) CEUS at 18 s shows early heterogeneous enhancement. (**C**) CEUS at 58 s shows reduced enhancement in part of the corresponding region compared with the early phase. (**D**) Axial T2-weighted image at the corresponding anatomical level. (**E**) High-b-value diffusion-weighted image (b = 2000 s/mm^2^) shows high signal in the right peripheral zone. (**F**) Corresponding apparent diffusion coefficient (ADC) map. The lesion was assigned PI-RADS 3, and the lesion ADC value was reported as 0.711 × 10^−3^ mm^2^/s. Biopsy showed prostate cancer with a Gleason score of 3 + 4 = 7 (ISUP Grade Group 2). White arrows indicate the lesion region.

The CEUS images were independently evaluated by two sonographers with at least 5 years of experience who were blinded to the patients’ clinical information and MRI scores. Assessment was qualitative and based on visual interpretation of enhancement patterns. CEUS positivity was defined as rapid early hyperenhancement followed by rapid washout relative to the surrounding or contralateral gland, presenting either focally or diffusely; all other enhancement patterns were classified as CEUS-negative. No time–intensity curves or quantitative perfusion parameters were analyzed. Figure 2 shows the ultrasound and MRI findings in a representative patient with a PI-RADS 3 lesion and biopsy-confirmed csPCa.

Any disagreement was reviewed by a third senior sonographer and resolved by consensus.

### 2.4. Prostate Biopsy and Histopathological Assessment

All patients underwent a 12-core prostate biopsy under transrectal ultrasound guidance. In patients with focal abnormal perfusion on CEUS, selected cores within the 12-core scheme were directed toward the corresponding suspicious region, while the remaining cores were obtained according to the systematic biopsy protocol. Biopsy specimens were evaluated by genitourinary pathologists, and Gleason scores and ISUP Grade Groups were assigned according to the International Society of Urological Pathology consensus criteria [21].

The study outcomes were biopsy-detected PCa and csPCa, with csPCa defined as PCa of ISUP Grade Group ≥ 2. For exploratory analyses, pathological findings were further classified as no PCa, ISUP Grade Group 1, ISUP Grade Groups 2–3, or ISUP Grade Groups 4–5.

### 2.5. Calculation and Selection of Inflammatory Markers

Ten inflammatory markers were calculated from peripheral blood counts obtained within 7 days before biopsy: the systemic immune-inflammation index (SII), NLR, systemic inflammation response index (SIRI), pan-immune-inflammation value (PIV), platelet-to-lymphocyte ratio (PLR), lymphocyte-to-monocyte ratio (LMR), eosinophil-to-lymphocyte ratio (ELR), basophil-to-lymphocyte ratio (BLR), neutrophil-to-monocyte ratio (NMR), and PMR. NLR was calculated as the absolute neutrophil count divided by the absolute lymphocyte count, and PMR as the platelet count divided by the absolute monocyte count. The remaining indices were calculated as follows: SII = P × N/L, SIRI = N × M/L, PIV = N × P × M/L, PLR = P/L, LMR = L/M, ELR = E/L, BLR = B/L, and NMR = N/M, where N, L, M, E, and B denote the absolute neutrophil, lymphocyte, monocyte, eosinophil, and basophil counts, respectively, and P denotes the platelet count.

Candidate markers were Z-score standardized and initially screened using 10-fold cross-validated least absolute shrinkage and selection operator logistic regression, with biopsy-detected PCa as the outcome [22]. Their discriminative performance was also assessed using individual receiver operating characteristic analyses. Candidate combined models were subsequently constructed and compared in terms of discrimination and relative model fit, leading to the selection of NLR and PMR for subsequent analyses.

### 2.6. Statistical Analysis

Interobserver agreement for the two sonographers’ individual binary CEUS classifications was assessed using unweighted Cohen’s κ with a 95% confidence interval and the percentage of concordant classifications, before consensus resolution. Continuous variables are presented as medians with interquartile ranges (IQRs), and categorical variables as frequencies and percentages. Between-group comparisons were performed using the Mann–Whitney U test, Pearson’s χ^2^ test, or Fisher’s exact test, as appropriate. Patients with missing variables were excluded from the corresponding analyses, and no missing-value imputation was performed.

Initial marker screening was performed using 10-fold cross-validated LASSO at λ_min and λ_1se. Two-marker models were subsequently constructed using NLR as the base marker. The independent effect of each additional marker, model fit, and discriminative performance were considered jointly, leading to the selection of NLR and PMR for subsequent analyses.

The NLR and PMR cutoff values were derived from separate ROC analyses using biopsy-detected PCa as the outcome, by maximizing the Youden index (sensitivity + specificity − 1). An NLR–PMR-positive phenotype was defined as either NLR reaching or exceeding its optimal cutoff or PMR reaching or falling below its optimal cutoff; the cutoff values used for classification were 2.495 and 386.402, respectively. A strict classification rule requiring both conditions was also evaluated. The diagnostic performance of each classification rule was assessed using sensitivity, specificity, predictive values, and the area under the receiver operating characteristic curve (AUC). Cutoff stability was evaluated using 1000 bootstrap resamples, with the median, IQR, and 95% empirical percentile interval reported.

Four joint subgroups were constructed according to CEUS status and the NLR–PMR phenotype, and the detection of PCa, csPCa, and tumors across ISUP Grade Groups was compared among these subgroups. The two NLR–PMR-negative subgroups were combined to form the low-risk stratum. Patients with an NLR–PMR-positive phenotype and negative CEUS were classified as intermediate risk, whereas those positive for both CEUS and NLR–PMR were classified as high risk. The Cochran–Armitage trend test was used to evaluate changes in outcome detection rates across risk strata and changes in the proportions of positive phenotypes across pathological categories. Within the NLR–PMR-positive subgroup, comparisons according to CEUS status were performed using two-sided Fisher’s exact tests with Holm correction for multiple comparisons. The 95% confidence intervals for the detection rates shown in the figures were calculated using the Clopper–Pearson exact binomial method.

Sequential logistic regression models were constructed separately for PCa and csPCa. M0 included age and PSAD; M1 added CEUS to M0; M2 added the continuous NLR–PMR score to M0; and M3 included both CEUS and the NLR–PMR score in addition to M0. The NLR–PMR score was generated from a logistic regression model with PCa as the outcome and standardized NLR and PMR as predictors. PSAD was scaled per 0.1 ng/mL/cm^3^ increase. Model discrimination was evaluated using the AUC and compared using the DeLong test [23]. Model fit was assessed using likelihood-ratio tests and the Akaike information criterion (AIC), and prediction error was evaluated using the Brier score.

Decision curve analysis (DCA) was used to compare the net benefit of M0 and M3 with the biopsy-all and biopsy-none strategies across threshold probabilities ranging from 2% to 30% [24]. Optimism was corrected using 1000 bootstrap resamples, with marker standardization, score derivation, and model fitting repeated in each resample, but without repeating candidate-marker selection [25]. This analysis was exploratory, and the selected threshold range should not be interpreted as recommended clinical thresholds for biopsy. An interaction model, M4, was constructed by adding the product term between CEUS and the continuous NLR–PMR composite score to M3, and Firth penalized logistic regression was used as a sensitivity analysis of the interaction effect [26]. In addition, an extended clinical adjustment analysis was performed by adding the free-to-total PSA ratio and PI-RADS score to the clinical baseline model.

The reported ROC curves and AUCs were based on apparent estimates. M0, M2, and M3 additionally underwent internal validation using 1000 bootstrap resamples to estimate optimism-corrected AUCs, Brier scores, calibration intercepts, and calibration slopes (Supplementary Table S6). Marker standardization, score derivation, and model fitting were repeated within each resample, whereas candidate-marker selection was not repeated. The net benefit estimates from DCA were also corrected for bootstrap optimism. No external validation was performed. All statistical analyses were conducted using R version 4.4.1. All tests were two-sided, and *p* < 0.05 was considered statistically significant.

## 3. Results

### 3.1. Patient Selection and Baseline Characteristics

Of the 2754 patients who underwent prostate biopsy during the study period, 418 met the eligibility criteria and were included in the final analysis (Figure 1). PCa was detected in 60 patients (14.4%), including 42 patients (10.0%) with csPCa. Patients with PCa were older and had higher PSAD and NLR values but lower PMR values than those without PCa (all *p* < 0.05). The proportions of patients with CEUS positivity and NLR–PMR positivity were also higher in the PCa group (60.0% vs. 41.9%, *p* = 0.009; 86.7% vs. 45.3%, *p* < 0.001; Table 1).

The two sonographers agreed on the CEUS classification in 371 of 418 patients (88.8%). Cohen’s κ was 0.773 (95% CI: 0.711–0.834).

### 3.2. Selection of Inflammatory Markers and Derivation of the NLR–PMR Phenotype

Among the 10 candidate inflammatory markers, NLR had the highest individual AUC (0.736, 95% CI: 0.665–0.806; Supplementary Table S1). LASSO retained six markers at λ_min and only NLR at λ_1se (Supplementary Figures S1 and S2). When PMR was added to NLR, PMR remained independently associated with PCa (OR = 0.68, 95% CI: 0.48–0.93, *p* = 0.023), and model fit improved (likelihood-ratio test *p* = 0.015), although the AUC did not increase (DeLong *p* = 0.876). The AIC values of the NLR + BLR and NLR + PMR models were similar (310.70 vs. 311.61), whereas the individual AUC of PMR was numerically higher than that of BLR (0.634 vs. 0.533). Considering these findings together with clinical interpretability, NLR and PMR were selected for subsequent analyses (Supplementary Table S2).

The optimal cutoff values for NLR and PMR were 2.495 and 386.402, respectively. Patients meeting either the elevated NLR or reduced PMR threshold were classified as NLR–PMR-positive, whereas those meeting neither condition were classified as NLR–PMR-negative. In 1000 bootstrap analyses, the median cutoff values for NLR and PMR were 2.49 and 386.60, respectively; their distributions are presented in Supplementary Table S3 and Supplementary Figure S3. The positive and negative groups included 214 and 204 patients, respectively. The corresponding detection rates were 24.3% and 3.9% for PCa and 17.8% and 2.0% for csPCa. All 15 ISUP Grade Group 4–5 cancers were NLR–PMR-positive. Diagnostic performance is presented in Table 2.

### 3.3. Risk Stratification Using the Combined CEUS and NLR–PMR Phenotype

The CEUS−/NLR–PMR−, CEUS+/NLR–PMR−, CEUS−/NLR–PMR+, and CEUS+/NLR–PMR+ groups included 120, 84, 112, and 102 patients, respectively. Overall differences in the detection rates of PCa, csPCa, and ISUP Grade Group 4–5 cancers across the four groups were statistically significant (all *p* < 0.001; Table 3). Among NLR–PMR-positive patients, the CEUS-positive group had higher detection rates for all three outcomes, with Holm-adjusted *p* values of 0.003, <0.001, and 0.014, respectively (Supplementary Table S4).

**Figure 3:**
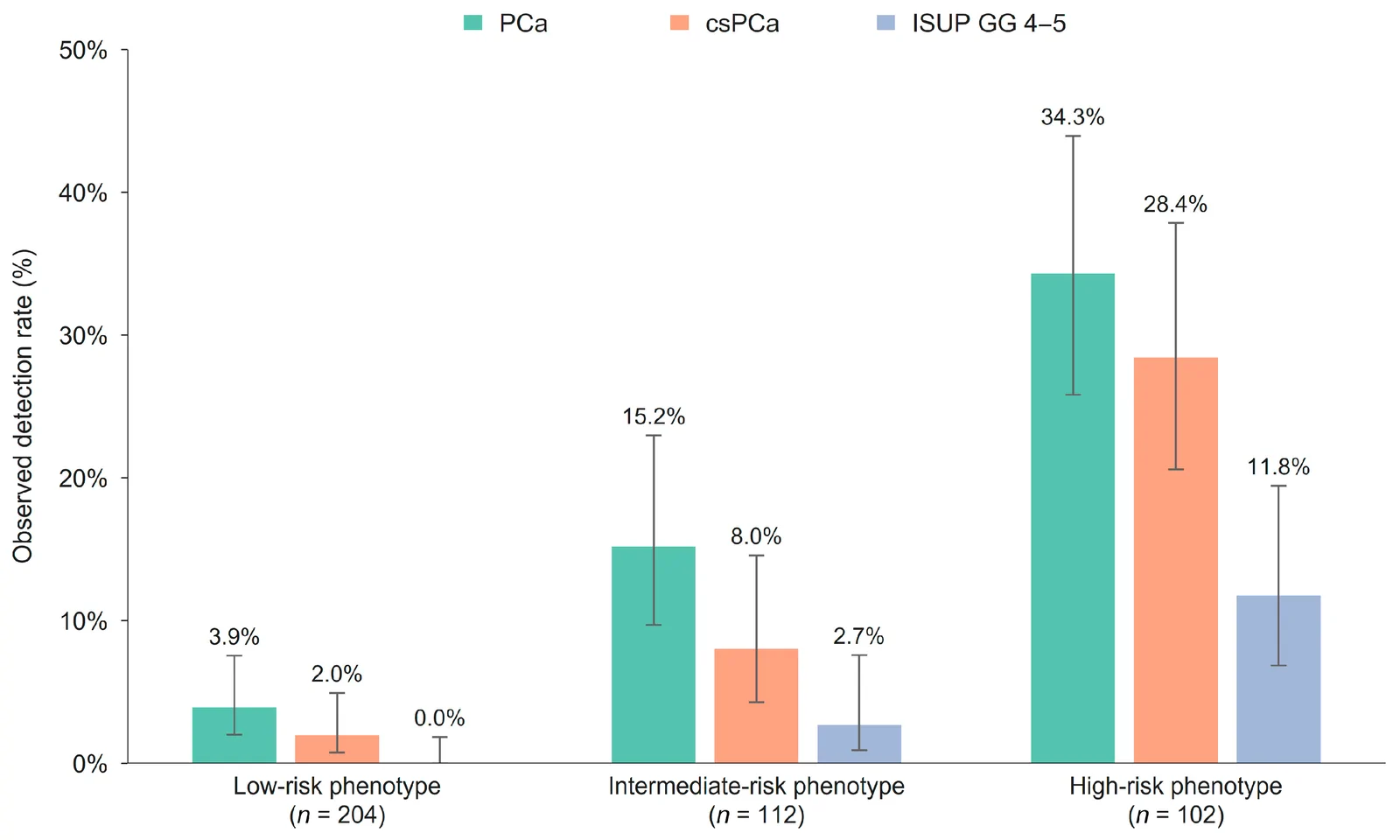
Detection rates across the three risk phenotypes. Error bars indicate 95% Clopper–Pearson confidence intervals. Low, intermediate, and high risk were defined as NLR–PMR negative, NLR–PMR positive/CEUS negative, and NLR–PMR positive/CEUS positive, respectively. All *p* for trend < 0.001.

For exploratory risk stratification, the two NLR–PMR-negative subgroups were combined to form the low-risk stratum, while the remaining two groups were classified as intermediate and high risk, respectively. Across the low-, intermediate-, and high-risk strata, the detection rates were 3.9%, 15.2%, and 34.3% for PCa; 2.0%, 8.0%, and 28.4% for csPCa; and 0%, 2.7%, and 11.8% for ISUP Grade Group 4–5 cancers, respectively (all *p* for trend < 0.001; Figure 3). The high-risk stratum comprised 24.4% of the cohort and included 69.0% of all csPCa cases and 80.0% of all ISUP Grade Group 4–5 cancers. The low-risk stratum comprised 48.8% of the cohort, with no ISUP Grade Group 4–5 cancers detected.

The proportions of CEUS positivity, NLR–PMR positivity, and dual positivity showed overall increasing trends across pathological categories (*p* for trend = 0.001, <0.001, and <0.001, respectively; Supplementary Table S5).

### 3.4. Predictive Performance of the Sequential Models

**Figure 4:**
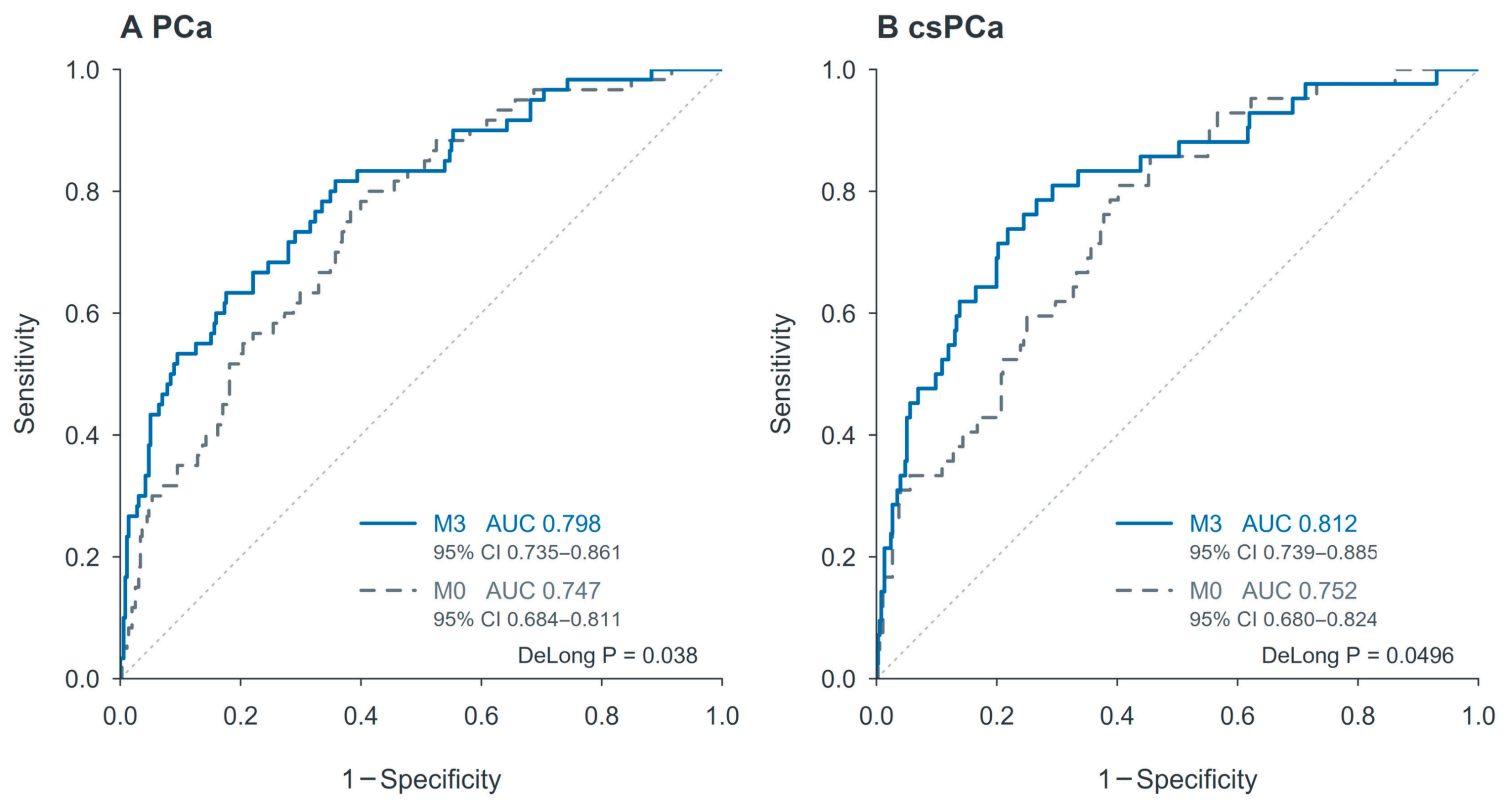
Receiver operating characteristic curves of the clinical and final combined models. ROC curves for (**A**) PCa and (**B**) csPCa. M0 included age and PSAD; M3 additionally included CEUS and the continuous NLR–PMR score. *p* values were obtained using paired DeLong tests. Results represent apparent performance without optimism correction.

The baseline model M0, comprising age and PSAD, yielded AUCs of 0.747 for PCa and 0.752 for csPCa. After addition of the continuous NLR–PMR score, the AUCs of M2 increased to 0.798 and 0.805, respectively (DeLong *p* = 0.025 and 0.026). With the further addition of CEUS, M3 yielded AUCs of 0.798 for PCa and 0.812 for csPCa, both higher than those of M0 (*p* = 0.038 and 0.0496). The differences in AUC between M3 and M2 did not reach statistical significance for either PCa (*p* = 0.953) or csPCa (*p* = 0.689). Model comparisons and the ROC curves for M0 and M3 are presented in Table 4 and Figure 4.

In the M3 model for csPCa, CEUS positivity remained independently associated with the outcome (OR = 2.26, 95% CI: 1.08–4.91, *p* = 0.033), and M3 showed improved model fit compared with M2 (likelihood-ratio test *p* = 0.030; Table 4).

After internal validation with 1000 bootstrap resamples, the optimism-corrected AUCs of M3 were 0.787 for PCa and 0.803 for csPCa, with calibration slopes of 0.940 and 0.936, respectively (Supplementary Table S6).

### 3.5. Exploratory Decision Curve Analysis

**Figure 5:**
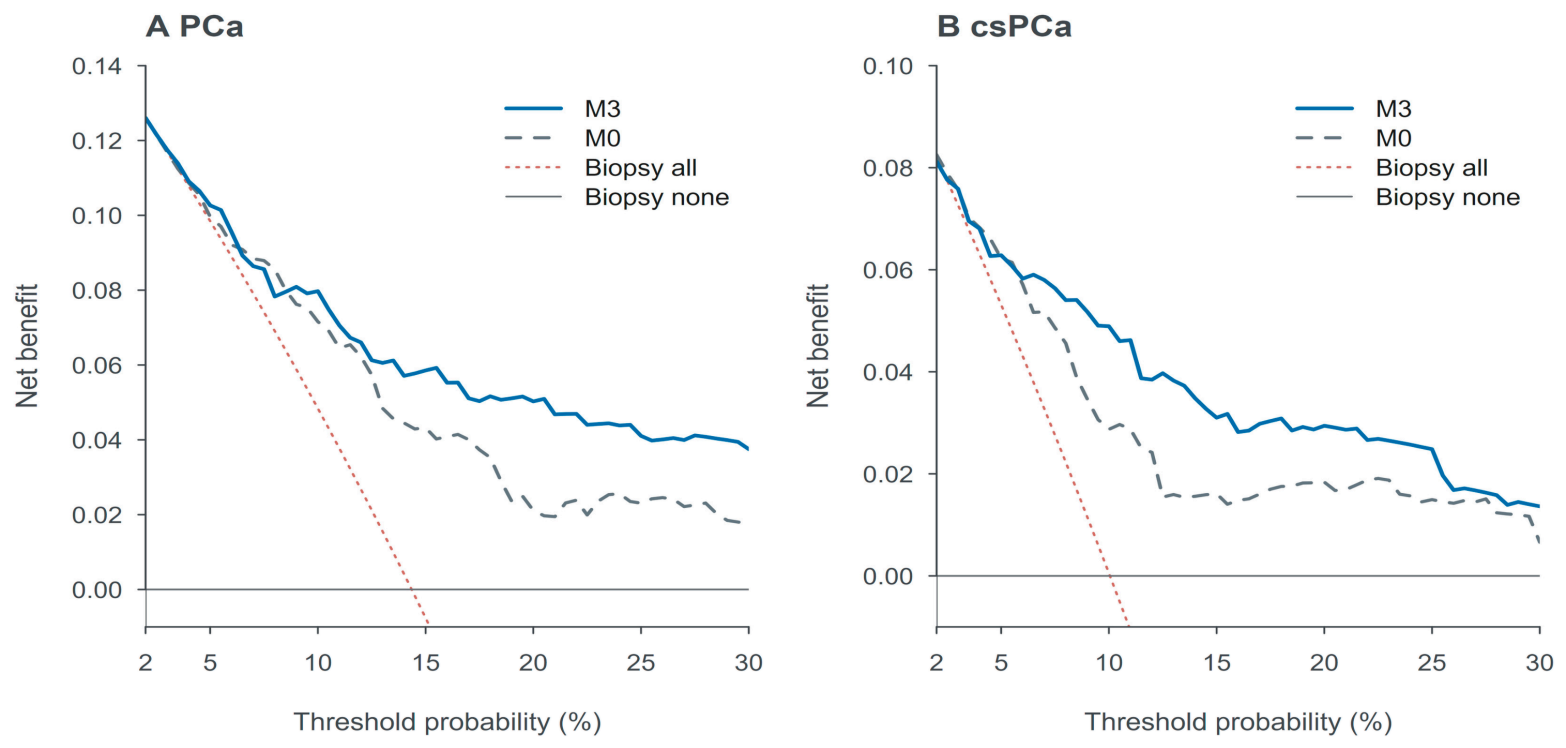
Bootstrap optimism-corrected decision curves for the clinical and combined models. Bootstrap optimism-corrected decision curves for (**A**) PCa and (**B**) csPCa. M0 included age and PSAD; M3 additionally included CEUS and the continuous NLR–PMR score. Corrections were based on 1000 bootstrap resamples. The evaluated threshold range of 2–30% was exploratory and does not represent recommended clinical thresholds. Note the different y-axis scales; negative net benefit below the plotting range is not shown.

Within the exploratory threshold probability range of 2–30%, M3 showed a more consistent net benefit advantage over M0 at approximately 10–30% after correction for optimism using 1000 bootstrap resamples. At some lower-threshold intervals, the curves crossed. These findings were observed for both PCa and csPCa (Figure 5). At threshold probabilities of 10%, 20%, and 30%, the corrected net benefit differences between M3 and M0 were 0.0082, 0.0292, and 0.0199 for PCa and 0.0202, 0.0110, and 0.0070 for csPCa, respectively.

### 3.6. Multivariable and Sensitivity Analyses

After further adjustment for the free-to-total PSA ratio and PI-RADS score, the association between CEUS and csPCa remained similar (OR = 2.28, 95% CI: 1.07–5.03, *p* = 0.035; Supplementary Table S7). Neither conventional logistic regression nor Firth regression provided clear evidence of an interaction between CEUS and the continuous NLR–PMR score for csPCa (*p* = 0.636 and 0.694, respectively; Supplementary Table S8).

## 4. Discussion

In patients with PSA levels of 4–20 ng/mL and PI-RADS scores ≤ 3, combined CEUS and NLR–PMR assessment identified groups with distinct cancer detection rates. NLR–PMR improved discrimination beyond age and PSAD. For csPCa, adding CEUS improved model fit (likelihood-ratio test *p* = 0.030), and CEUS positivity remained associated with the outcome after adjustment (OR = 2.26, 95% CI: 1.08–4.91). Among NLR–PMR-positive patients, csPCa detection rates were 28.4% with positive CEUS and 8.0% with negative CEUS. These findings support the potential of the combined assessment to refine pre-biopsy risk stratification in this clinically uncertain population.

The full model achieved AUCs of 0.798 for PCa and 0.812 for csPCa, both higher than those of the baseline model comprising age and PSAD. Liu et al. previously reported that a model combining CEUS and PI-RADS showed good discriminative performance in patients with PSA levels of 4–10 ng/mL [11]. The present study further focused on patients with low-suspicion MRI findings and explored the predictive and stratification value of incorporating hematological information. The CADMUS study also found that the clinically significant tumors detected by multiparametric ultrasound, which included CEUS, did not completely overlap with those detected by mpMRI, although this finding cannot be attributed to CEUS alone [27]. In the present study, adding CEUS to the continuous-score model did not significantly improve AUC, whereas CEUS status was still associated with different detection risks among NLR–PMR-positive patients. Together with the observed subgroup risk separation, these findings support further evaluation of CEUS as a supplementary risk-assessment tool.

The performance of inflammatory markers has varied across study populations. In 276 patients with PI-RADS 3 lesions, Kayar et al. reported associations between NLR, PIV, SIRI, and PCa detection, with SIRI showing relatively better discrimination [28]. In a separate two-center cohort, adding SII and NLR increased the AUC for PI-RADS 3 lesions, but the difference was not statistically significant [29]. These findings indicate that an association between a marker and an outcome does not necessarily translate into a consistent improvement in model performance. Salciccia et al. also found substantial heterogeneity in the prognostic evidence for inflammatory ratios [15]. Prognostic findings and pre-biopsy diagnostic performance concern different clinical settings and require separate interpretation. In the present study, NLR and PMR were retained after comparisons of candidate markers to evaluate their contribution within the defined PSA and MRI population. Gokce et al. reported that NLR was even higher in patients with prostatitis than in those with PCa, further indicating that these markers should not be directly equated with a tumor-related immune state [30].

Lower PMR was independently associated with PCa in our cohort. A lower ratio can reflect a higher monocyte count, a lower platelet count, or both. Relative monocyte predominance provides a possible biological context, given the role of monocyte/macrophage-related processes in the tumor microenvironment. Loberg et al. demonstrated that CCL2 blockade reduced macrophage infiltration, microvascular density, and tumor growth in a prostate cancer xenograft model [31]. Circulating PMR does not directly measure intratumoral macrophage abundance or activity. Studies combining individual blood-cell counts with tissue immune markers could clarify the basis of this association.

The double-negative subgroup did not have the lowest PCa detection rate, a finding that requires cautious interpretation. The detection rate was 1.2% in the CEUS+/NLR–PMR− subgroup, compared with 5.8% in the double-negative subgroup; however, only one PCa case was detected in the former subgroup. Sparse events and random variation may therefore have contributed substantially to this nonmonotonic distribution. Previous studies have reported that some cases of benign prostatic hyperplasia and chronic inflammation may exhibit abnormal enhancement resembling that of malignant lesions, providing another possible explanation for this finding [12]. However, because enhancement patterns were not directly matched with benign histopathological findings in this study, these results should not be interpreted as indicating a protective effect of CEUS positivity or an ability of NLR–PMR to exclude false-positive CEUS findings. Combining the two NLR–PMR-negative subgroups simplified risk presentation but did not establish that their risks were equivalent.

The three-tier classification revealed substantial differences in cancer risk among patients sharing the same broad PSA and MRI eligibility criteria. The low-risk stratum comprised 48.8% of the cohort and had a csPCa detection rate of 2.0%, whereas the high-risk stratum comprised 24.4% and included 69.0% of all csPCa cases and 80.0% of Grade Group 4–5 cancers. This concentration of clinically significant disease suggests that the combined assessment may help identify patients who warrant greater attention during pre-biopsy evaluation. Within the NLR–PMR-positive subgroup, CEUS further separated patients with csPCa detection rates of 28.4% and 8.0%, providing additional information when blood-based assessment indicated elevated risk. Previous studies combining MRI and PSAD have similarly emphasized risk-adapted biopsy triage integrating complementary risk information [32]. These findings give the combined phenotype a clinically interpretable role and support evaluating its contribution to individualized biopsy decisions. The low-risk stratum still contained csPCa cases, so the classification requires prospective evaluation before it can guide biopsy deferral. The interaction analyses provided no clear evidence of an interaction, and the observed risk gradient should not be interpreted as evidence of a biological synergistic effect.

Exploratory DCA complemented these findings from a decision-making perspective. DCA uses threshold probabilities to reflect the trade-off between missed diagnoses and unnecessary interventions [24]. Within the evaluated range of 2–30%, the net benefit advantage of M3 over M0 was more consistent at approximately 10–30%; the curves crossed over some lower-threshold intervals. A threshold probability represents the estimated cancer risk at which biopsy would be considered worthwhile. For example, a 10% csPCa threshold means considering biopsy when estimated csPCa risk reaches or exceeds 10%. Lower thresholds place greater emphasis on avoiding missed cancers and favor biopsy in more patients, leaving less opportunity for additional information to change decisions. The observed advantage at approximately 10–30% suggests a potentially more favorable balance between cancer detection and unnecessary biopsies when thresholds in this range are chosen. At csPCa thresholds of 10%, 20%, and 30%, the corrected net benefit differences between M3 and M0 corresponded to net biopsy reduction equivalents of 18.2, 4.4, and 1.6 per 100 patients, respectively. These threshold-weighted estimates illustrate the potential benefit and do not represent observed reductions in biopsy procedures. Threshold selection depends on the outcome of interest, clinical circumstances, and patient preferences. This range describes the comparative advantage observed in this cohort and does not establish recommended biopsy thresholds. The magnitude and clinical importance of net benefit differences require assessment through independent validation and prospective evaluation of actual biopsy decisions [33].

The hematological component uses routine pre-biopsy blood-count data. When CEUS findings are already available, the combined assessment can use existing information without requiring another scan or clinic visit. When CEUS would be an additional examination, patients with unresolved biopsy decisions would be a relevant population for prospective evaluation. The observed subgroup risk separation provides a rationale for examining this focused application. Costs and additional clinic attendance were not assessed in this study. Future comparisons with the current diagnostic pathway should consider biopsy decisions, missed csPCa, additional examinations and visits, and costs.

This study has several limitations. First, the retrospective single-center design may have introduced selection bias. Biopsy was performed following clinical assessment, so the cohort represents patients selected for both CEUS and biopsy within the PSA and MRI criteria. Clinical selection may affect the distribution of cancer risk and the observed detection rates. PPV and NPV depend on disease prevalence; at comparable sensitivity and specificity, higher prevalence generally increases PPV and decreases NPV. Detailed PSA and MRI data were not collected for patients who did not undergo CEUS. Consequently, the total number of otherwise eligible biopsy patients and the proportion receiving CEUS could not be determined. Interpretation in other settings should consider local referral criteria and disease prevalence.

The 60 PCa and 42 csPCa events limit the precision and stability of multivariable estimates and four-group comparisons. Although CEUS positivity remained associated with csPCa after adjustment (OR = 2.26, 95% CI: 1.08–4.91, *p* = 0.033), the confidence interval is compatible with a broad range of effect sizes. The limited number of csPCa events may contribute to this uncertainty. Marker selection, cutoff determination, and model evaluation were performed in the same cohort. Candidate-marker selection was not repeated during internal validation or DCA correction, leaving the possibility of residual optimism.

Biopsy pathology may be affected by sampling error and grade underestimation. Comparisons with radical prostatectomy specimens have shown that underdetection and grade underestimation may persist even when systematic and targeted biopsy are combined [8]. In our study, suspicious CEUS regions could influence core placement within the 12-core scheme, which may also affect the observed relationship between CEUS and biopsy detection. Reliable information on subsequent radical prostatectomy and postoperative pathology was unavailable; the number undergoing surgery and performance against surgical pathology could therefore not be assessed. The reported outcomes relate to biopsy-detected PCa and csPCa.

CEUS assessment was qualitative, and time–intensity curves or quantitative perfusion parameters were not analyzed. Quantitative analysis could provide additional information on enhancement kinetics. A single complete blood count could not capture temporal changes in inflammatory markers. NLR and PMR are non-specific markers that may be influenced by prostatitis, urinary tract infections, and antibiotic use. Patients with histological chronic inflammation were retained, and clinical infection status at blood sampling and recent antibiotic use were not systematically collected. Consequently, a sensitivity analysis addressing these factors could not be performed, and residual confounding may affect the observed associations and model performance. The extent of this influence cannot be determined from the available data.

Independent external validation was unavailable. Transferability may be influenced by the distribution of PI-RADS scores, csPCa prevalence, patient characteristics, biopsy strategies, and pathological reference standards. Differences in MRI equipment and protocols, CEUS equipment and acquisition techniques, contrast agents, and reader experience may also affect performance. We have planned prospective, multicenter external validation to assess discrimination, calibration, and potential net benefit in patients meeting the original eligibility criteria. Consecutive enrollment and documentation of CEUS use and biopsy referral reasons will be important for assessing selection effects; recalibration may be needed in new settings.

## 5. Conclusions

In this exploratory single-center cohort of patients with PSA levels of 4–20 ng/mL and PI-RADS scores ≤ 3, NLR–PMR improved discrimination for PCa and csPCa, and CEUS further differentiated csPCa risk among NLR–PMR-positive patients. The combined assessment identified strata with progressively increasing cancer detection rates, providing preliminary evidence for individualized pre-biopsy risk stratification. Its value for guiding biopsy decisions requires prospective, multicenter external validation.

## Figures and Tables

**Table 1:** Baseline clinical, imaging, and inflammatory characteristics of the study cohort.

Characteristics	Non-PCa (*n* = 358)	PCa (*n* = 60)	*p* Value
Age, years	67 (62–72)	70 (66.5–74)	0.005
Total PSA, ng/mL	8.85 (6.64–11.89)	10.27 (7.52–12.85)	0.007
Prostate volume, mL	61.50 (43.50–82.73)	43.54 (32.17–53.99)	<0.001
Free-to-total PSA ratio	0.178 (0.125–0.232)	0.134 (0.106–0.175)	<0.001
PSAD, ng/mL/cm^3^	0.144 (0.103–0.213)	0.235 (0.183–0.347)	<0.001
NLR	2.06 (1.60–2.80)	3.04 (2.47–4.42)	<0.001
PMR	481.12 (397.86–627.20)	392.44 (283.58–539.33)	<0.001
CEUS status, *n* (%)			0.009
Negative	208 (58.1)	24 (40.0)	
Positive	150 (41.9)	36 (60.0)	
NLR-PMR phenotype, *n* (%)			<0.001
Negative	196 (54.7)	8 (13.3)	
Positive	162 (45.3)	52 (86.7)	
PI-RADS score, *n* (%)			0.443
2	35 (9.8)	4 (6.7)	
3	323 (90.2)	56 (93.3)	

Note: Data are presented as median (interquartile range) for continuous variables and number (percentage) for categorical variables. Continuous variables were compared using the Mann–Whitney U test, and categorical variables were compared using Pearson’s χ^2^ test without continuity correction. Abbreviations: CEUS, contrast-enhanced ultrasound; IQR, interquartile range; NLR, neutrophil-to-lymphocyte ratio; PCa, prostate cancer; PI-RADS, Prostate Imaging Reporting and Data System; PMR, platelet-to-monocyte ratio; PSA, prostate-specific antigen; PSAD, prostate-specific antigen density.

**Table 2:** Diagnostic performance of NLR, PMR, and their combined decision rules for PCa and csPCa detection.

Strategy	Sensitivity, %	Specificity, %	PPV, %	NPV, %	Accuracy, %	AUC (95% CI)
Panel A. PCa detection						
NLR alone	75.0	69.3	29.0	94.3	70.1	0.736 (0.665–0.806)
PMR alone	50.0	77.4	27.0	90.2	73.4	0.634 (0.554–0.714)
Either-positive rule	86.7	54.7	24.3	96.1	59.3	0.707 (0.657–0.758)
Double-positive rule	38.3	91.9	44.2	89.9	84.2	0.651 (0.588–0.715)
Panel B. csPCa detection						
NLR alone	76.2	67.3	20.6	96.2	68.2	0.729 (0.644–0.814)
PMR alone	54.8	76.6	20.7	93.8	74.4	0.660 (0.573–0.747)
Either-positive rule	90.5	53.2	17.8	98.0	56.9	0.718 (0.667–0.770)
Double-positive rule	40.5	90.7	32.7	93.2	85.6	0.656 (0.579–0.732)

Note: Classification used NLR ≥ 2.495 and PMR ≤ 386.402. The either-positive rule required either criterion, whereas the double-positive rule required both. PCa-derived cutoffs were applied to csPCa. Individual-marker AUCs were based on continuous values and rule-based AUCs on binary classifications. csPCa was defined as Grade Group ≥ 2. Abbreviations: AUC, area under the receiver operating characteristic curve; CI, confidence interval; csPCa, clinically significant prostate cancer; NLR, neutrophil-to-lymphocyte ratio; NPV, negative predictive value; PCa, prostate cancer; PMR, platelet-to-monocyte ratio; PPV, positive predictive value.

**Table 3:** Distribution of biopsy outcomes according to the combined CEUS/NLR-PMR phenotype.

Combined Phenotype	*n*	Non-PCa, *n* (%)	PCa, *n* (%)	csPCa, *n* (%)	ISUP GG 1, *n* (%)	ISUP GG2–3, *n* (%)	ISUP GG4–5, *n* (%)
CEUS−/NLR-PMR−	120	113 (94.2)	7 (5.8)	4 (3.3)	3 (2.5)	4 (3.3)	0 (0.0)
CEUS+/NLR-PMR−	84	83 (98.8)	1 (1.2)	0 (0.0)	1 (1.2)	0 (0.0)	0 (0.0)
CEUS−/NLR-PMR+	112	95 (84.8)	17 (15.2)	9 (8.0)	8 (7.1)	6 (5.4)	3 (2.7)
CEUS+/NLR-PMR+	102	67 (65.7)	35 (34.3)	29 (28.4)	6 (5.9)	17 (16.7)	12 (11.8)

Note: Data are presented as *n* (%), calculated within each phenotype group. NLR–PMR positivity was defined as NLR ≥ 2.495 or PMR ≤ 386.402. csPCa was defined as ISUP GG ≥ 2. Overall group differences were assessed using Pearson’s χ^2^ or Fisher’s exact test, as appropriate (all *p* < 0.001). Abbreviations: CEUS, contrast-enhanced ultrasound; csPCa, clinically significant prostate cancer; GG, Grade Group; NLR, neutrophil-to-lymphocyte ratio; PCa, prostate cancer; PMR, platelet-to-monocyte ratio.

**Table 4:** Predictive performance and model comparisons for PCa and csPCa.

Outcome	Model	AUC (95% CI)	Model Comparison	DeLong *p*	LRT *p*
PCa	M0	0.747 (0.684–0.811)	Reference		
	M1	0.757 (0.693–0.822)	M1 vs. M0	0.496	0.058
	M2	0.798 (0.736–0.859)	M2 vs. M0	0.025	<0.001
	M3	0.798 (0.735–0.861)	M3 vs. M0	0.038	<0.001
	M3	0.798 (0.735–0.861)	M3 vs. M2	0.953	0.301
csPCa	M0	0.752 (0.680–0.824)	Reference		
	M1	0.781 (0.708–0.854)	M1 vs. M0	0.238	0.007
	M2	0.805 (0.738–0.873)	M2 vs. M0	0.026	<0.001
	M3	0.812 (0.739–0.885)	M3 vs. M0	0.0496	<0.001
	M3	0.812 (0.739–0.885)	M3 vs. M2	0.689	0.030

Note: M0 included age and PSAD; M1 added CEUS to M0; M2 added the continuous NLR–PMR score to M0; and M3 added CEUS to M2. DeLong tests compared AUCs, whereas likelihood-ratio tests compared model fit. Apparent AUCs are presented; corresponding optimism-corrected performance estimates are reported in Supplementary Table S6.

## Data Availability

The data supporting the findings of this study are not publicly available due to privacy or ethical restrictions. Requests for access to the data should be directed to the corresponding authors.

## Supplementary Materials

The following supporting information can be downloaded at https://www.mdpi.com/article/10.3390/diagnostics16213680/s1, Supplementary Methods; Table S1: Diagnostic performance of individual inflammatory markers for PCa detection; Table S2: Comparative performance of NLR-based candidate inflammatory models; Table S3: Bootstrap distributions of the optimal cutoff values for NLR and PMR; Table S4: Outcomes stratified by CEUS status among NLR–PMR-positive patients; Table S5: Trends in CEUS and NLR–PMR phenotypes across pathological categories; Table S6: Bootstrap internal validation of the prediction models; Table S7: Association of CEUS with PCa and csPCa in extended multivariable models; Table S8: Interaction between CEUS and the continuous NLR–PMR score; Figure S1: LASSO coefficient profiles of the candidate inflammatory markers; Figure S2: Ten-fold cross-validation for LASSO tuning-parameter selection; Figure S3: Bootstrap distributions of the optimal NLR and PMR cutoff values.
