# Supplementary material for: Contrast-Enhanced Ultrasound Combined with the NLR–PMR Phenotype for Prostate Cancer Prediction and Risk Stratification in Patients with PSA 4–20 ng/mL and PI-RADS ≤ 3
Source: Diagnostics (Basel). 2026 Nov 7;16(21):3680. doi: 10.3390/diagnostics16213680 (PMC13763353; doi:10.3390/diagnostics16213680)
Supplement: Supplementary file 1 [file diagnostics-16-03680-s001.zip › diagnostics-4644476-supplementary.pdf]

Supplementary Material

Contrast-Enhanced Ultrasound Combined with the NLR–PMR Phenotype for Prostate Cancer Prediction and Risk Stratification in Patients with PSA 4–20 ng/mL and PI-RADS ≤ 3

Supplementary Tables S1–S8 and Supplementary Figures S1–S3

Supplementary Methods

Inflammatory-marker selection

All candidate inflammatory markers were Z-standardized before modeling. Ten-fold cross-validated LASSO logistic regression was evaluated at λ_min and λ_1se. Dual-marker logistic models were then constructed by adding each LASSO-retained candidate to NLR. The additional marker was evaluated using the Wald test, nested-model improvement using the likelihood-ratio test, and discrimination using the AUC and paired DeLong test. AIC and BIC were used to compare relative model fit.

Cutoff stability

The optimal NLR and PMR cutoffs were determined from the PCa ROC curves using the maximum Youden index. Cutoff stability was evaluated using 1000 patient-level bootstrap resamples. The bootstrap median, IQR, and 2.5th–97.5th percentile range were summarized.

Internal validation

M0, M2, and M3 were internally validated using 1000 bootstrap resamples. NLR and PMR were retained as fixed selected predictors; within each resample, marker standardization and the continuous NLR–PMR score were reconstructed and each prediction model was refitted. Optimism-corrected AUC, Brier score, calibration intercept, and calibration slope were estimated. Candidate-marker selection was not repeated.

Sensitivity analyses

Extended models additionally adjusted for the free-to-total PSA ratio and PI-RADS score. Potential interaction between CEUS and the continuous NLR–PMR score was assessed in conventional logistic regression, with Firth penalized logistic regression used as a sensitivity analysis. All analyses were performed using R version 4.4.1.

Supplementary Table S1. Diagnostic performance of individual inflammatory markers for PCa detection

Inflammatory marker	AUC (95% CI)	*p* value
SII	0.690 (0.614–0.766)	<0.001
NLR	0.736 (0.665–0.806)	<0.001
SIRI	0.732 (0.662–0.802)	<0.001
PIV	0.696 (0.619–0.773)	<0.001
PLR	0.565 (0.484–0.647)	0.105
LMR	0.681 (0.604–0.757)	<0.001
ELR	0.521 (0.435–0.606)	0.610
BLR	0.533 (0.456–0.609)	0.420
NMR	0.622 (0.548–0.695)	0.003
PMR	0.634 (0.554–0.714)	<0.001

Note: Each marker was evaluated using ROC analysis. For markers inversely associated with PCa, the ROC direction was reversed so that larger AUC values indicated better discrimination. *p* values were obtained using the Mann–Whitney U test comparing patients with and without PCa.

Abbreviations: AUC, area under the receiver operating characteristic curve; BLR, basophil-to-lymphocyte ratio; CI, confidence interval; ELR, eosinophil-to-lymphocyte ratio; LMR, lymphocyte-to-monocyte ratio; NLR, neutrophil-to-lymphocyte ratio; NMR, neutrophil-to-monocyte ratio; PCa, prostate cancer; PIV, pan-immune-inflammation value; PLR, platelet-to-lymphocyte ratio; PMR, platelet-to-monocyte ratio; SII, systemic immune-inflammation index; SIRI, systemic inflammation response index.

Supplementary Table S2. Comparative performance of NLR-based candidate inflammatory models

Model	AUC (95% CI)	AIC	BIC	Wald *p*	Likelihood-ratio *p*	DeLong *p*
NLR alone	0.736 (0.665–0.806)	315.53	323.60	Reference	Reference	Reference
NLR + SII	0.734 (0.663–0.804)	317.18	329.28	0.567	0.551	0.592
NLR + PMR	0.732 (0.659–0.805)	311.61	323.72	0.023	0.015	0.876
NLR + PIV	0.735 (0.665–0.806)	317.05	329.15	0.462	0.487	0.929
NLR + ELR	0.732 (0.662–0.801)	316.79	328.90	0.372	0.390	0.560
NLR + BLR	0.726 (0.656–0.795)	310.70	322.81	0.016	0.009	0.709

Note: Wald *p* evaluates the additional marker in each dual-marker model. Likelihood-ratio and DeLong *p* values compare each dual-marker model with the NLR-only model. All markers were Z-standardized before fitting. Lower AIC and BIC values indicate better relative fit.

Abbreviations: AIC, Akaike information criterion; AUC, area under the receiver operating characteristic curve; BIC, Bayesian information criterion; BLR, basophil-to-lymphocyte ratio; CI, confidence interval; ELR, eosinophil-to-lymphocyte ratio; NLR, neutrophil-to-lymphocyte ratio; PIV, pan-immune-inflammation value; PMR, platelet-to-monocyte ratio; SII, systemic immune-inflammation index.

Supplementary Table S3. Bootstrap distributions of the optimal cutoff values for NLR and PMR

Marker	Original cutoff	Bootstrap median	IQR	95% range
NLR	2.49	2.49	2.49–2.54	2.25–2.81
PMR	386.40	386.60	385.81–399.25	298.08–569.92

Note: Cutoff stability was evaluated using 1000 bootstrap resamples. The 95% range represents the 2.5th–97.5th percentiles. Cutoffs are displayed in rounded form; phenotype classifications used the unrounded values.

Abbreviations: IQR, interquartile range; NLR, neutrophil-to-lymphocyte ratio; PMR, platelet-to-monocyte ratio.

Supplementary Table S4. Outcomes stratified by CEUS status among NLR–PMR-positive patients

Outcome	CEUS negative (*n* = 112)	CEUS positive (*n* = 102)	Fisher *p*	Holm-adjusted *p*
PCa	17 (15.2%)	35 (34.3%)	0.0014	0.0027
csPCa	9 (8.0%)	29 (28.4%)	<0.001	<0.001
ISUP GG 4–5	3 (2.7%)	12 (11.8%)	0.0137	0.0137

Note: Data are *n* (%). Two-sided Fisher exact tests were used, with Holm adjustment across the three outcomes.

Abbreviations: CEUS, contrast-enhanced ultrasound; csPCa, clinically significant prostate cancer; GG, Grade Group; NLR, neutrophil-to-lymphocyte ratio; PCa, prostate cancer; PMR, platelet-to-monocyte ratio.

Supplementary Table S5. Trends in CEUS and NLR–PMR phenotypes across pathological categories

Indicator	Non-PCa (n = 358)	ISUP GG 1 (n = 18)	ISUP GG 2–3 (n = 27)	ISUP GG 4–5 (n = 15)	p for trend
CEUS positive	150 (41.9%)	7 (38.9%)	17 (63.0%)	12 (80.0%)	0.001
NLR–PMR positive	162 (45.3%)	14 (77.8%)	23 (85.2%)	15 (100.0%)	<0.001
CEUS+/NLR–PMR+	67 (18.7%)	6 (33.3%)	17 (63.0%)	12 (80.0%)	<0.001

Note: Data are *n* (%), with percentages calculated within each pathological category. Cochran–Armitage tests assessed linear trend across ordered categories. NLR–PMR positivity required NLR ≥2.49 or PMR ≤386.40; displayed cutoffs are rounded and classifications used the unrounded values. The combined phenotype required both CEUS positivity and NLR–PMR positivity.

Abbreviations: CEUS, contrast-enhanced ultrasound; GG, Grade Group; NLR, neutrophil-to-lymphocyte ratio; PCa, prostate cancer; PMR, platelet-to-monocyte ratio.

Supplementary Table S6. Bootstrap internal validation of the prediction models

Outcome	Model	Apparent AUC	Corrected AUC	Apparent Brier	Corrected Brier	Corrected calibration intercept	Corrected calibration slope
PCa	M0	0.747	0.745	0.111	0.113	0.000	0.981
PCa	M2	0.798	0.792	0.098	0.102	−0.001	0.956
PCa	M3	0.798	0.787	0.098	0.102	−0.001	0.940
csPCa	M0	0.752	0.750	0.081	0.083	0.006	0.981
csPCa	M2	0.805	0.800	0.076	0.080	0.005	0.953
csPCa	M3	0.812	0.803	0.074	0.078	0.004	0.936

Note: Internal validation used 1000 bootstrap resamples. NLR and PMR were fixed selected predictors. Within each resample, the continuous NLR–PMR score was reconstructed and the prediction models were refitted; LASSO screening and candidate-marker selection were not repeated. All resamples produced valid estimates. M0 included age and PSAD; M2 added the continuous NLR–PMR score; M3 added CEUS to M2.

Abbreviations: AUC, area under the receiver operating characteristic curve; CEUS, contrast-enhanced ultrasound; csPCa, clinically significant prostate cancer; NLR, neutrophil-to-lymphocyte ratio; PCa, prostate cancer; PMR, platelet-to-monocyte ratio; PSAD, prostate-specific antigen density.

Supplementary Table S7. Association of CEUS with PCa and csPCa in extended multivariable models

Outcome	Model	CEUS OR (95% CI)	*p* value
PCa	E1	1.71 (0.93–3.15)	0.084
PCa	E3	1.38 (0.72–2.65)	0.329
csPCa	E1	2.58 (1.25–5.56)	0.012
csPCa	E3	2.28 (1.07–5.03)	0.035

Note: E1 included age, PSAD, free-to-total PSA ratio, PI-RADS score, and CEUS. E3 additionally included the continuous NLR–PMR score. ORs represent the effect of CEUS positivity.

Abbreviations: CEUS, contrast-enhanced ultrasound; CI, confidence interval; csPCa, clinically significant prostate cancer; NLR, neutrophil-to-lymphocyte ratio; OR, odds ratio; PCa, prostate cancer; PI-RADS, Prostate Imaging Reporting and Data System; PMR, platelet-to-monocyte ratio; PSAD, prostate-specific antigen density.

Supplementary Table S8. Interaction between CEUS and the continuous NLR–PMR score

Outcome	Conventional logistic *β*	*p* value	Firth logistic *β*	*p* value
PCa	−2.681	0.322	−2.293	0.356
csPCa	1.125	0.636	0.859	0.694

Note: *β* is the regression coefficient for the CEUS × continuous NLR–PMR interaction term. Firth penalized logistic regression was used as a sensitivity analysis.

Abbreviations: CEUS, contrast-enhanced ultrasound; csPCa, clinically significant prostate cancer; NLR, neutrophil-to-lymphocyte ratio; PCa, prostate cancer; PMR, platelet-to-monocyte ratio.

Supplementary Figure S1. LASSO coefficient profiles of the candidate inflammatory markers

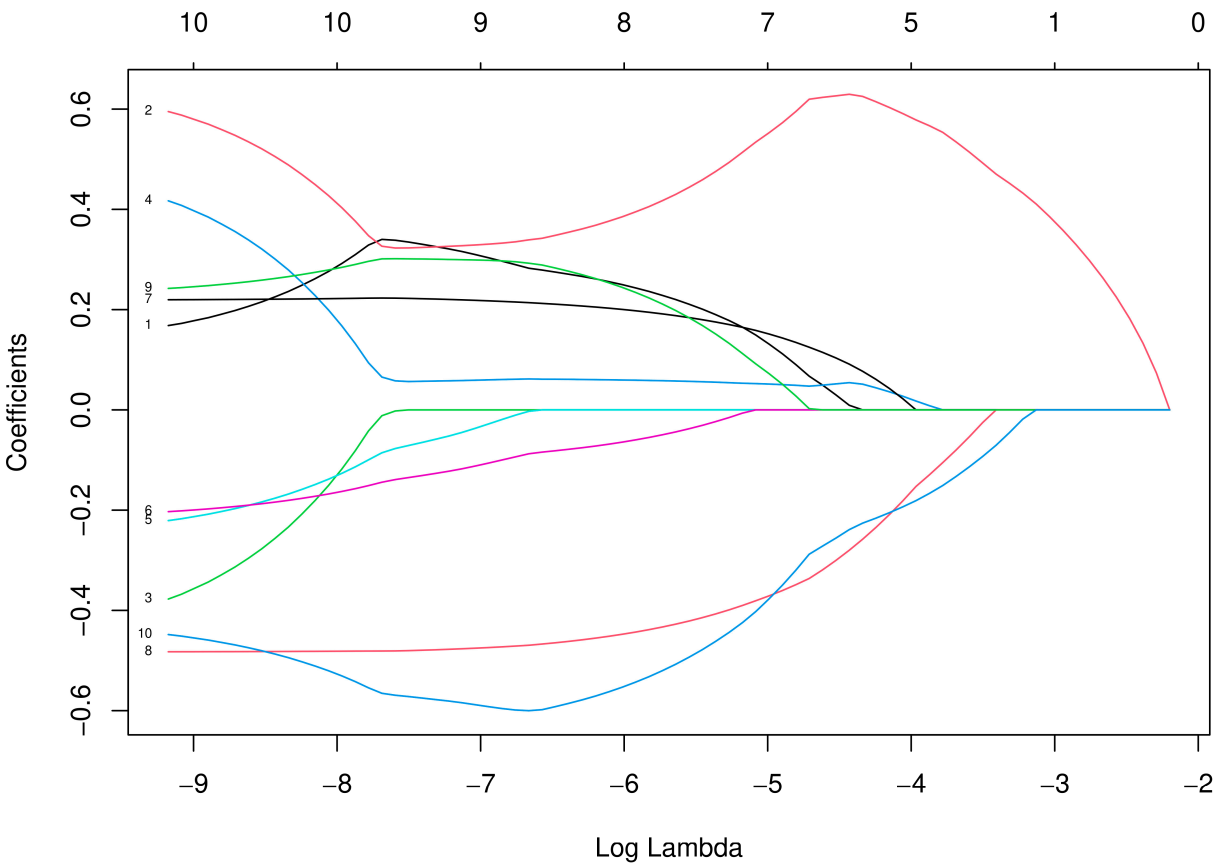

Coefficient trajectories are shown across the log-lambda sequence. Numbers at the top indicate the number of nonzero coefficients. Predictor labels correspond to the input order: 1, SII; 2, NLR; 3, SIRI; 4, PIV; 5, PLR; 6, LMR; 7, ELR; 8, BLR; 9, NMR; and 10, PMR.

Supplementary Figure S2. Ten-fold cross-validation for LASSO tuning-parameter selection

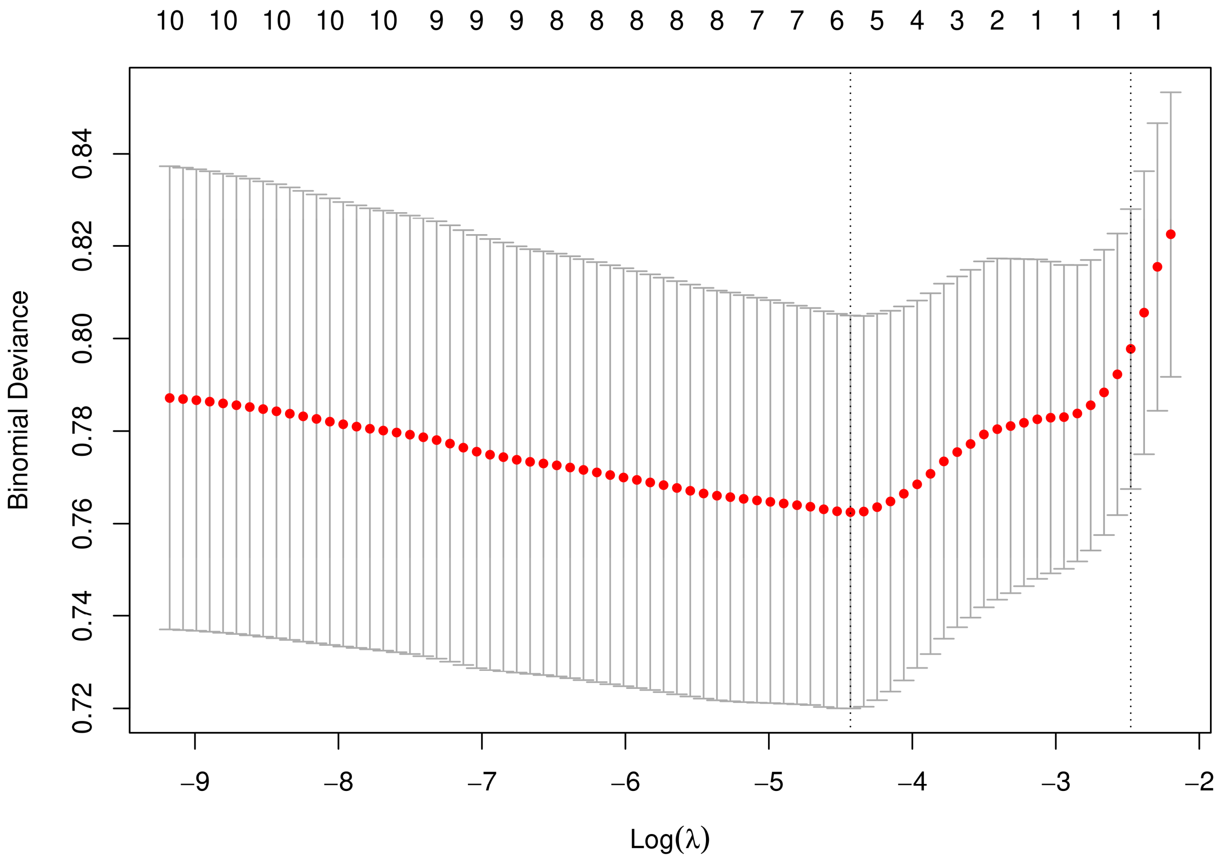

Red points show the mean cross-validated binomial deviance and error bars show one standard error. The two vertical dotted lines indicate λ_min and λ_1se. Numbers at the top indicate the number of nonzero coefficients.

Supplementary Figure S3. Bootstrap distributions of the optimal NLR and PMR cutoff values

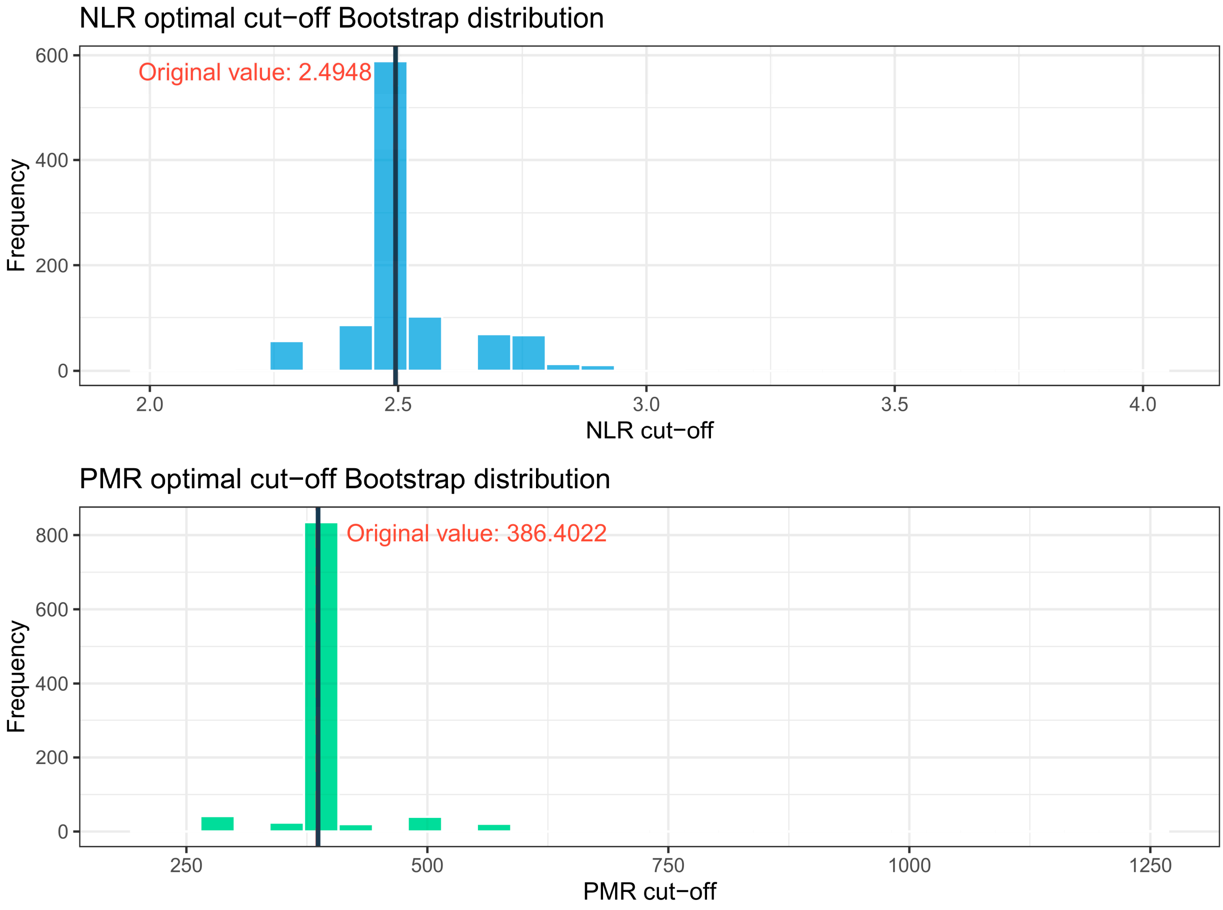

Histograms show the optimal cutoff values obtained in 1000 bootstrap resamples. Vertical lines indicate the original-sample cutoffs for NLR (2.4948) and PMR (386.4022).
